# OA01 Multifaceted triggers, diagnostic and management hurdles in hemophagocytic lymphohistiocytosis in a young immunosuppressed adult with systemic juvenile idiopathic arthritis

**DOI:** 10.1093/rap/rkae117.001

**Published:** 2024-11-05

**Authors:** Nadia Tripp, Elizabeth Coulson

**Affiliations:** Northumbria Healthcare, Northumberland, United Kingdom; Northumbria Healthcare, Northumberland, United Kingdom

## Abstract

**Introduction:**

We present a case of a 17 year old female with systemic JIA (generally well controlled for a long period on sc Tocilizumab), who presented unwell with fevers, sore throat and significant lymphadenopathy. She was initially treated for tonsillar sepsis in the context of immunosuppression, but clinically deteriorated. Further tests suggested she had a life-threatening condition Haemophagocytic Lymphohistiocytosis (HLH). She received immunosuppressive treatment and her clinical condition improved. Further testing showed EBV was the likely trigger for HLH and treatment was tailored to this, whilst liaising with haematology. She made a good recovery but required nearly a month in hospital.

**Case description:**

• 17 year old female seen in clinic 5/3/24 and reported hip pain suggestive of flare up of JIA. Plan from clinic was USS guided hip injection as previously had inflammation at this site. Reported clinically well in clinic and had returned from holiday in Lanzarote 24/2/24. Bloods all normal and reported compliance with Tocilizumab. Did not attend for USS guided injection.

• 26/3/24 - contacted rheumatology complaining of sore throat, stated clinically well in self and when called back had seen GP who had seen tonsillar exudate and given antibiotics. Advised to withhold Tocilizumab and attend A&E if worsened.

• 30/3/24 presented to A&E unwell with fevers and cervical lymphadenopathy. Diagnosed with sepsis (and bloods suggestive of this with raised CRP, normal FBC). Sepsis treatment escalated with further investigation when did not improve.

• 2/4/24 - rheumatology called and bloods now showed pancytopenia (Hb 92, Plt 58, WCC 2.5) with coagulopathy, CRP 123, ESR <3. Further tests suggested for H score (triglycerides 2.5, fibrinogen <1, AST 350, USS 20cm splenomegaly). Clinical condition continued to deteriorate with NEWS 10 (HR 215 bpm). Despite H score not fully being back team recognised this was likely HLH and advised treatment with IVMP and ITU transfer.

• 3/4/24 - dramatic clinical improvement following IVMP. Disclosed not been compliant with Tocilizumab when on ITU - so thought flare of sJIA triggering HLH and advised IV Tocilizumab.

• 4/4/24 - ferritin finally back at 32,000ng/mL, also EBV PCR 88,000 suggesting EBV triggered HLH. Rituximab advised by haematology. IVIg also given. Clinical state continued to improve.

• 6/4/24 - transferred to tertiary centre where continued to improve and was treated with Anakinra, further Rituximab infusion and reducing prednisolone.

• 23/4/24 - discharged with follow up from haematology and rheumatology and plan to re-start IV Tocilizumab to ensure compliance. Gene panel for primary HLH also sent but results still awaited.

**Discussion:**

• Complexity in context of possible sepsis and existing immunosuppression. In this case tonsillar exudate and lymphadenopathy was present, making team think this was all infective initially. Flare up of systemic JIA was also considered - especially when we realised she had been ‘under-dosed’ with Tocilizumab due to reduced compliance. Discussion also regarding difficulties in management/compliance some young adult patients.

• EBV infection was thought to be the trigger (we consulted experts on this) due to such a high EBV PCR, such marked cervical lymphadenopathy and demographic of young person. There are 4 ways EBV can trigger HLH - as new infection, as reactivation of old infection (as in this case), as chronic infection and as EBV-associated with malignancy. This is interesting discussion and learning point.

• When called our department on 26/3/24 we wondered if we should have done blood tests to compare to admission ones (which didn’t show pancytopenia which was present only a few days into admission) as may have alerted us to trend/drop earlier.

• Difficulties in diagnosing HLH promptly in acute medical setting of a District General Hospital (DGH) - e.g. 2 day delay in getting ferritin back despite phone calls to lab multiple times. Changes to take place and reduce delays in HLH diagnosis and improve management (raising awareness, Trust guidelines, HLH bundle on requesting system as plans).

• Interesting ESR very low in HLH and is used as a marker of HLH activity in primary HLH patients. ESR was <3 while CRP 123 and ferritin 32,000.

• Marked tachycardia of 215bpm prior to ITU transfer - discussion regarding best place to look after HLH patients (most agree ITU is best and evidence does suggest tachycardia is precursor to cardiac arrest in these cases so important to monitor and recognise cardiac events can occur).

**Key learning points:**

• Always think of HLH in any unwell, febrile patient with low cell counts.

• Involve seniors, get H score and escalate early so treatment can be started promptly.

• It is not unusual to need several modalities of immunosuppression - in this case the patient had IVMP, Tocilizumab, IVIg, Rituximab and Anakinra.

• EBV is a common trigger of HLH and should be considered in context of young adults presenting with HLH. It is further complicated in the context of immunosuppression where the EBV could be new infection, reactivation of old infection, chronic infection or even EBV associated with malignancy. EBV-triggered HLH is the trickiest kind of HLH to treat. Rituximab is recommended and liaising closely with haematology.

• Infection can trigger HLH and this case was complicated by initially presenting to GP with sore throat, tonsillar exudate, being advised to withhold Tocilizumab by rheumatology and treating for sepsis on medical ward before HLH considered.

• Challenges exist with engaging young adults with chronic health conditions. In this case the patient initially reported compliance with Tocilizumab in clinic, but later disclosed to me in ITU that she took it very rarely. When very unwell she was frightened and promised to take medication as prescribed, but when recovered was less keen to engage with healthcare and did not attend some appointments.

• Patients with systemic JIA are more likely to develop HLH than patients with other rheumatic diseases. This patient’s young age has also led to specialists suggesting gene panel for primary HLH is sent in case there is a genetic cause (results pending at time of abstract submission).

• Increased awareness of HLH in our Trust - 2 education sessions delivered by myself on this (to ITU and rheumatology departments), plan to embed in Trust guidelines, awareness of national MDT and help from experts is available.

